# Hyperosmotic Stress Induces Unconventional Autophagy Independent of the Ulk1 Complex

**DOI:** 10.1128/MCB.00024-19

**Published:** 2019-07-29

**Authors:** Naoki Tamura, Shun Kageyama, Masaaki Komatsu, Satoshi Waguri

**Affiliations:** a Department of Anatomy and Histology, Fukushima Medical University School of Medicine, Fukushima, Japan; b Department of Biochemistry, Niigata University Graduate School of Medical and Dental Sciences, Niigata, Japan; c Department of Physiology, Juntendo University Graduate School of Medicine, Tokyo, Japan

**Keywords:** Ulk1, autophagy, hyperosmotic stress, mTOR

## Abstract

Autophagy is considered an adaptive mechanism against hyperosmotic stress. Although the process has been reported to be triggered by the inhibition of mTORC1, the precise downstream mechanisms remain elusive. Here, we demonstrate that hyperosmotic-stress-induced autophagy is different from conventional macroautophagy in mouse embryonic fibroblasts (MEFs) and human T24 cells. Our results indicated that cytoplasmic puncta for the isolation membrane markers WIPI2 and Atg16L increased after hyperosmotic stress. They were found to partially colocalize with puncta for a selective autophagy substrate, SQSTM1/p62, and were shown to be diminished by inhibitors of phosphatidylinositol 3-kinase (PI3K) or by knockdown of human Vps34 (hVps34), a component of PI3K. In addition, flux assays showed that SQSTM1/p62 and NcoA4 were degraded by the lysosomal pathway. Surprisingly, Ulk1, which is essential for starvation-induced macroautophagy, remained inactivated under hyperosmotic stress, which was partially caused by mTOR activity. Accordingly, the Ulk1 complex was not nucleated under hyperosmotic stress. Finally, autophagy proceeded even in MEFs deficient in *RB1CC1/FIP200* or *Atg13*, which encode components of the Ulk1 complex. These data suggest that hyperosmotic-stress-induced autophagy represents an unconventional type of autophagy that bypasses Ulk1 signaling.

## Abstract (precis)

Autophagy is considered an adaptive mechanism against hyperosmotic stress. Although the process has been reported to be triggered by the inhibition of mTORC1, the precise downstream mechanisms remain elusive. Here, we demonstrate that hyperosmotic-stress-induced autophagy is different from conventional macroautophagy in mouse embryonic fibroblasts (MEFs) and human T24 cells.

## INTRODUCTION

Hyperosmotic stress is considered one of the important environmental stimuli that mammalian cells inevitably experience. It is primarily exemplified by epithelial cells in renal tubules and by the alimentary tract, which are exposed to osmotic fluctuations in urine and digested materials, respectively (1–3). In addition, plasma hypertonicity is also present in the context of pathological conditions, such as diabetes (4), and in therapeutic interventions using mannitol, such as in the case of intracranial hypertension (5). When naive cells, including cultured cells, are exposed to hyperosmotic stress, rapid cell shrinkage occurs in a few minutes, followed by the gradual influx of ions and water to increase the cell volume. This cell shrinkage initiates changes in macromolecular crowding, ionic strength, and the mechanical/chemical properties of the lipid bilayer (2). Because the majority of cellular components are affected, severe cases can lead to cell death. Therefore, to survive under such harsh conditions, cells possess various adaptive responses, including the activation of multiple signal transduction pathways, such as those involving p38 and Jun N-terminal protein kinase (JNK) mitogen-activated protein kinases (MAPKs) (6, 7); unique responses to DNA damage, mitochondrial dysfunction, and ROS production (1, 8, 9); cytoskeletal remodeling (10–12); the suppression of vesicular trafficking (9, 13–15); protein ubiquitination and aggregation (16–19); and autophagic degradation (10, 20).

Macroautophagy (referred to here as autophagy) is a lysosomal degradation system in which an isolation membrane (IM) (also known as a phagophore) sequesters a portion of the cytoplasm to become an autophagosome, which then fuses with lysosomes in order to degrade its contents (21). This process is triggered by several stimuli, including nutrient and energy depletion, which generally leads to the suppression of mechanistic target of rapamycin (mTOR) activity. The uncoordinated 51-like kinase 1 (Ulk1) complex is then activated and nucleated as the autophagosome formation site. Atg9-containing vesicles and the class III phosphatidylinositol 3-kinase (PI3K) complex, which produces the phosphatidylinositol 3-phosphate (PI3P)-rich precursor structure known as the omegasome, are recruited. The PI3P-binding protein, the WD repeat proteins interacting with phosphoinositide (WIPI), and Atg2 then promote the expansion of the IM, which eventually closes to become the autophagosome. The Atg12-Atg5-Atg16L complex is also recruited to the IM, producing the lipidated form of microtubule-associated protein light chain 3 (LC3-II) on the membrane, which is considered to be involved in the closure and fusion of the autophagosome with the lysosome and/or the selective engulfment of large substrates through binding to autophagy receptors (21).

Recent studies have shown that hyperosmotic stress induces acute autophagy for purposes of survival in the porcine renal proximal tubule-like cell line LLC-PK_1_ and two human cancer cell lines, namely, HCT116 and HeLa cells (10, 20). This conclusion was based on the results of the increased degradation of long-lived proteins, the upregulation of autophagic flux, and PI3K-dependent formation of LC3- and Atg12-positive puncta (10). In addition, studies have shown that mTOR is suppressed while the AMP-activated protein kinase (AMPK) is upregulated with treatments involving mannitol or sorbitol (20, 22–24). These findings appear to strengthen a mechanistic model of hyperosmotic-stress-induced autophagy that is mediated by mTOR/AMPK. However, in this study, using mouse embryonic fibroblasts (MEFs) and T24 cells, we found that Ulk1 was inactivated under conditions of hyperosmotic stress, while puncta for IM markers were increased. Moreover, autophagy was still induced in *RB1CC1/FIP200* (referred to here as *FIP200*)- or *Atg13*-deficient MEFs. Therefore, collectively, the data presented strongly suggest that Ulk1-independent autophagy occurs under hyperosmotic stress.

## RESULTS

### Hyperosmotic stress induces autophagy in MEFs and T24 cells.

**FIG 1:**
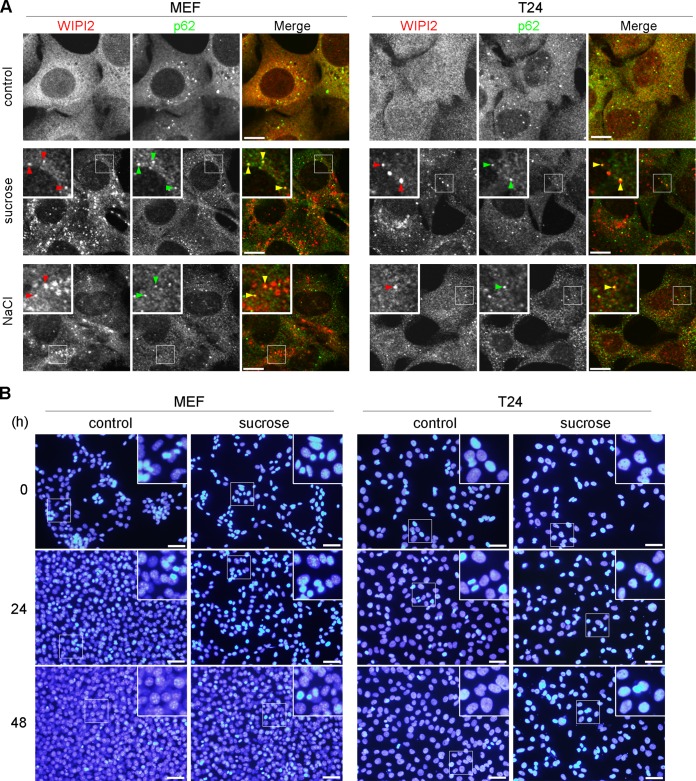
Hyperosmotic stress induces autophagosomal structures in MEFs and T24 cells. (A) Wild-type MEFs and T24 cells were cultured in DMEM (control) or DMEM with 0.8 M sucrose or 0.4 M NaCl for 30 min. The cells were fixed and double immunolabeled for WIPI2 (red) and p62 (green). The boxed regions are magnified in the insets. Punctate structures positive for both signals (yellow) are indicated by arrowheads. Bars, 10 μm. (B) After being subjected to osmotic stress with 0.8 M sucrose for 1 h, the cells were further cultured for 24 or 48 h. They were then fixed with 4% paraformaldehyde, and the nuclei were stained with Hoechst 3334. Images were acquired with a fluorescence microscope. The boxed regions are magnified in the insets. Note that no apparent nuclear fragmentation is observed and that the smaller condensed structures are chromosomes in a cell division phase. Bars, 50 μm.

**FIG 2:**
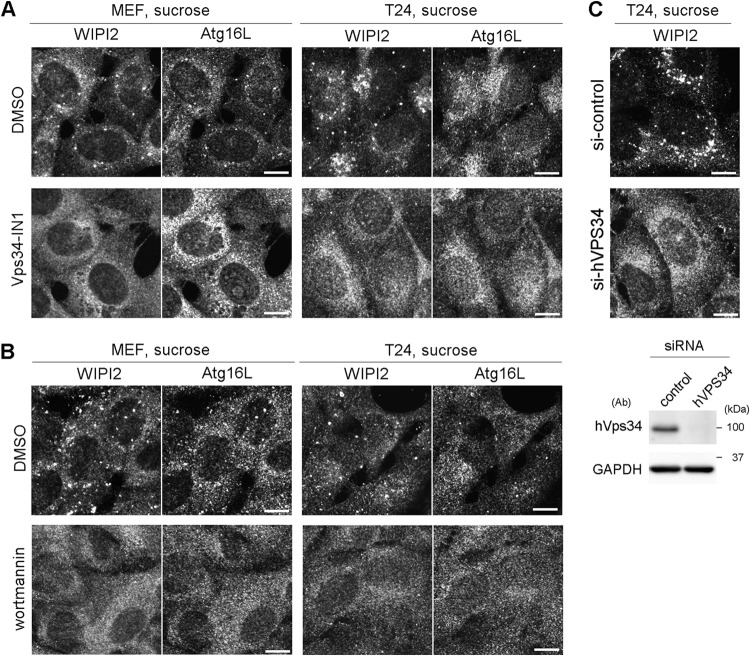
WIPI2/Atg16L punctum formation after hyperosmotic stress is dependent on Vps34. (A) Wild-type MEFs and T24 cells were cultured in DMEM with dimethyl sulfoxide (DMSO) or 200 nM Vps34-IN1 for 60 min and shifted to medium containing 0.8 M sucrose with the same concentration of DMSO or Vps34-IN1. After incubation for 30 min, they were fixed and double immunolabeled for Atg16L and WIPI2. Bars, 10 μm. (B) Wild-type MEFs and T24 cells were cultured in DMEM with DMSO or 1 μM wortmannin for 120 min and shifted to medium containing 0.8 M sucrose with the same concentration of DMSO or wortmannin. After incubation for 30 min, they were fixed and double immunolabeled for WIPI2 and Atg16L. Bars, 10 μm. (C) T24 cells transfected with siRNAs for control (si-control) or hVPS34 (si-hVPS34) were shifted to hyperosmotic medium for 30 min. They were then fixed and immunolabeled for WIPI2. Depletion of hVPS34 by the siRNAs was confirmed by Western blotting (bottom). GAPDH was used as a loading control. The antibodies (Ab) used and molecular weights are shown. Bars, 10 μm.

**FIG 3:**
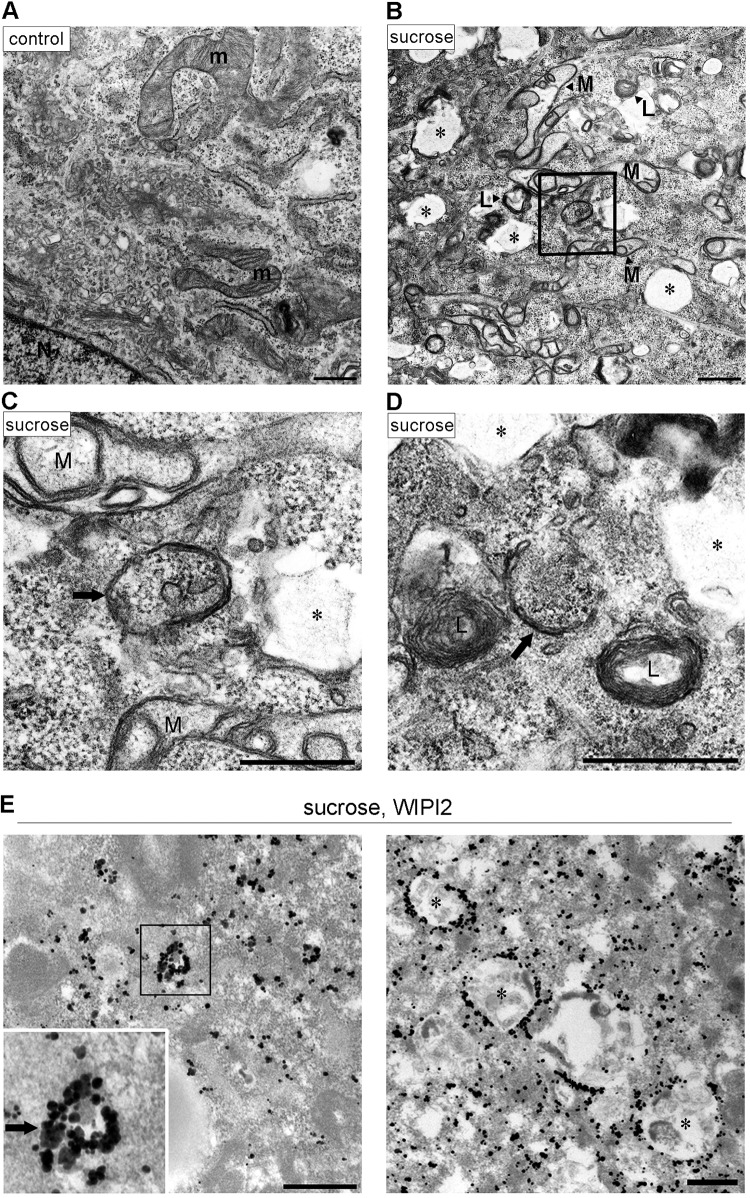
Electron microscopic analyses for autophagosomal structures and WIPI2 localization under hyperosmotic stress. (A to D) Wild-type MEFs were cultured in DMEM with (B to D) or without (A) 0.8 M sucrose for 1 h and then fixed for EM. The boxed region in panel B is shown enlarged in panel C. (E) Wild-type MEFs were cultured in DMEM with 0.8 M sucrose for 30 min and then fixed with 4% paraformaldehyde alone (left) or 4% paraformaldehyde-0.01% glutaraldehyde (right) for immuno-EM using anti-WIPI2 antibody. Note that after hyperosmotic stress, large endosome-like structures (asterisks), lysosome-like profiles (L), and degenerated mitochondria (M) increased. m, mitochondria in the control; N, nucleus. Bars, 0.5 μm.

Hyperosmotic-stress-induced autophagy has been previously demonstrated in various cell lines, including LLC-PK1, HCT116, and HeLa cells (10, 20). Therefore, we first reevaluated the phenomenon in MEFs and the T24 cell line, which is derived from human urinary bladder carcinoma. By using immunofluorescence (IF) microscopy, WIPI2, a marker protein for omegasomes and IMs (25), appeared as a cytoplasmic punctate signal after treatment with 0.8 M sucrose or 0.4 M NaCl for 30 min (Fig. 1A). Although the concentration of sucrose was higher than those reported previously (0.1 to 0.5 M) (20, 22–24), we confirmed that this concentration did not cause apparent cell death but transiently suppressed cell growth (Fig. 1B). By double immunofluorescence, a substrate of selective autophagy, SQSTM1/p62 (referred to here as p62), was also found as cytoplasmic punctate structures under normal culture conditions and became partially colocalized with WIPI2 under hyperosmotic stress (Fig. 1A). Another IM marker, Atg16L, was also found to colocalize with WIPI2, and their formation of puncta was suppressed by both specific and broad class III PI3K inhibitors, namely, Vps34-IN1 and wortmannin, respectively (Fig. 2A and B). We also confirmed, by utilizing small interfering RNA (siRNA) experiments in T24 cells, that the nucleation of WIPI2 was dependent on human Vps34 (hVps34), a component of the PI3K complex (Fig. 2C). These findings suggest that this type of autophagy is dependent on the presence of PI3P on membranes and the fact that punctum formation is not just nonspecific aggregation. By using electron microscopy (EM), hyperosmotic conditions were observed to cause an increase in the numbers of large endosome-like structures, deformed mitochondria, and multilamellar lysosome-like structures. In addition, autophagosome/IM-like profiles, which typically possess double membranes, were occasionally observed (Fig. 3A to D). Moreover, immuno-EM revealed that WIPI2 localizes on autophagosome/IM-like profiles containing engulfed materials and large endosome-like structures containing membranous fragments (Fig. 3E). These results suggest the occurrence of hyperosmotic-stress-induced autophagy, as reported previously, but with morphological alterations of many organelles other than autophagy-related structures.

**FIG 4:**
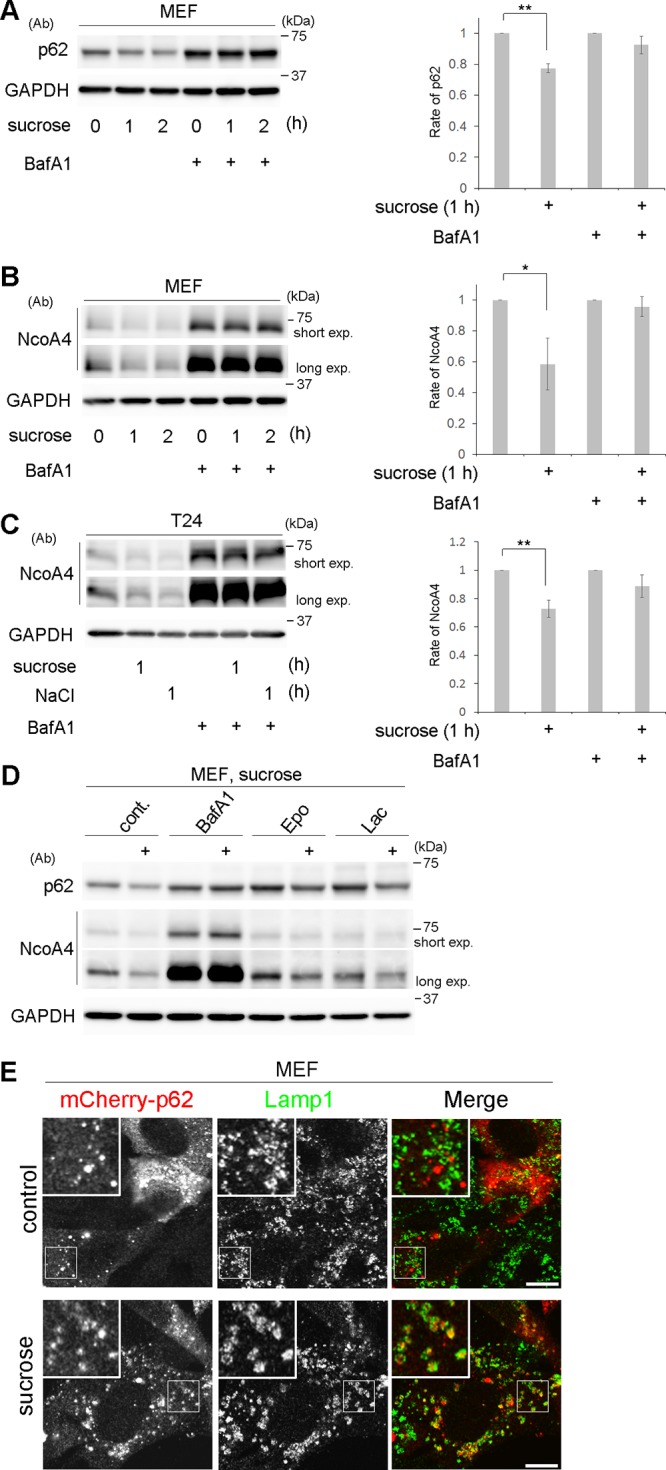
Hyperosmotic stress induces lysosomal degradation of p62 and NcoA4. (A to C) Wild-type MEFs (A and B) and T24 cells (C) were cultured with or without 100 nM bafilomycin A1 (BafA1) for 16 h and then shifted to DMEM with 0.8 M sucrose or 0.4 M NaCl for 0, 1, or 2 h, as indicated. The lysates were analyzed by Western blotting using antibodies against p62 (A), NcoA4 (B and C), or GAPDH. Molecular weights are indicated on the right. Bands of short and long exposure (exp.) are shown for NcoA4. The experiments were repeated 3 (A), 4 (B), and 5 (C) times. Band intensities were quantified, and ratios to the control value are plotted on the right. Statistical significances were determined by Student's *t* test. *, *P < *0.05; **, *P < *0.01. (D) Wild-type MEFs were cultured without (cont.) or with 100 nM BafA1 or two proteasome inhibitors, 5 μM epoxomicin (Epo) and 10 μM lactacystin (Lac), for 6 h. They were then shifted to DMEM with 0.8 M sucrose for 1 h (+). The lysates were analyzed by Western blotting using antibodies (Ab) against p62, NcoA4, or GAPDH, as indicated. Molecular weights are shown on the right. (E) MEFs transfected with mCherry-p62 (red) were cultured in DMEM (control) or DMEM with 0.8 M sucrose for 1 h and then fixed and immunostained for Lamp1 (green). The boxed regions are magnified in the insets. Bars, 10 μm.

We then monitored the degradation of p62 and another selective substrate or cargo receptor, NcoA4, by using flux assays. In MEFs, the two proteins were significantly decreased under hyperosmotic conditions, which was blocked by treatment with bafilomycin A1 (Fig. 4A and B) but not by proteasome inhibitors (Fig. 4D). The same results were obtained for NcoA4 in T24 cells (Fig. 4C). Furthermore, mCherry-tagged p62 was often detected inside Lamp1-positive lysosomes after hyperosmotic stress (Fig. 4E), indicating that p62 is transported to lysosomes during this degradation process. Together, these results indicate that selective autophagy is induced under hyperosmotic stress in MEFs and T24 cells.

### Ulk1 remains phosphorylated at Ser757 under hyperosmotic stress, which is partially mediated by mTORC1 activity.

**FIG 5:**
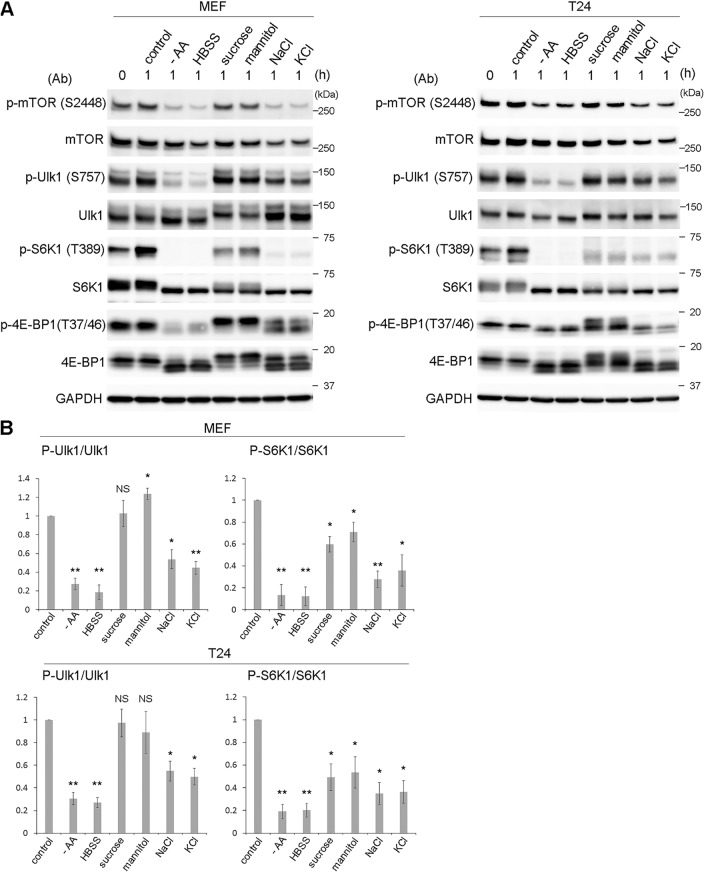
Ulk1 is phosphorylated at S757, while mTOR kinase activity is partially suppressed under hyperosmotic stress. (A) Wild-type MEFs and T24 cells were cultured with amino acid-free DMEM (−AA) or Hanks balanced salt solution (HBSS) or DMEM with 0.8 M sucrose, 0.8 M mannitol, 0.4 M NaCl, or 0.4 M KCl for 1 h. A sample without the cultures was also included in the leftmost lane (0 h). Cells were lysed for Western blot analyses using antibodies against phosphorylated mTOR (S2448), mTOR, phosphorylated Ulk1 (S575), Ulk1, phosphorylated S6K1 (T389), S6K1, phosphorylated 4E-BP1 (T37/46), 4E-BP1, and GAPDH as a loading control. Molecular weights are indicated on the right. (B) The experiments were repeated 3 times. Band intensities were quantified, and the ratio of the phosphorylated form to total protein was calculated, which was then compared to the control value. Statistical significance between the control and each condition was determined by Student's *t* test. *, *P < *0.05; **, *P < *0.01; NS, not significant.

The Ulk1 complex regulates starvation-induced autophagy via the mTOR complex 1 (mTORC1) pathway, which has not been investigated in hyperosmotic-stress-induced autophagy. Therefore, we next tested the signaling states of mTORC1 and Ulk1 by Western blotting (WB) with anti-phosphoprotein antibodies under hyperosmotic conditions. Ulk1 is known to be phosphorylated by mTORC1 at Ser757; thus, it is dephosphorylated under nutrient deprivation. As expected, amino acid deprivation caused a decrease in phosphorylated mTOR (p-mTOR) (S2448), which led to the drastic dephosphorylation of its substrates, ribosomal S6 kinase 1 (S6K1) (T389), eukaryotic translation initiation factor 4E-binding protein 1 (4E-BP1) (T37/46), and Ulk1 (S757) (Fig. 5). The responses to hyperosmotic stress differed according to the osmolytes used. Stress induced by nonionic osmolytes, such as sucrose or mannitol, did not significantly change the S2448 phosphorylation of mTOR and caused the phosphorylation of the three substrates differently. In this process, phosphorylated Ulk1 (p-Ulk1) was unchanged or, rather, increased when mannitol was added; phosphorylated S6K1 (p-S6K1) was partially but significantly reduced; and 4E-BP1 appeared to be phosphorylated (p-4E-BP1) differently than was observed under control conditions (Fig. 5). On the other hand, stress induced with ionic osmolytes, such as NaCl or KCl, reduced p-mTOR (S2448) and caused partial but significant reduction of p-S6K1 and p-Ulk1. p-4E-BP1 was reduced and shifted to smaller forms (Fig. 5). In summary, although p-S6K1 was significantly reduced under hyperosmotic stress, as reported previously, the level was not as low as that observed with amino acid deprivation. In addition, the alterations were not necessarily correlated with those of p-Ulk1 or p-4E-BP1, and importantly, p-Ulk1 was not decreased after treatment with nonionic osmolytes.

**FIG 6:**
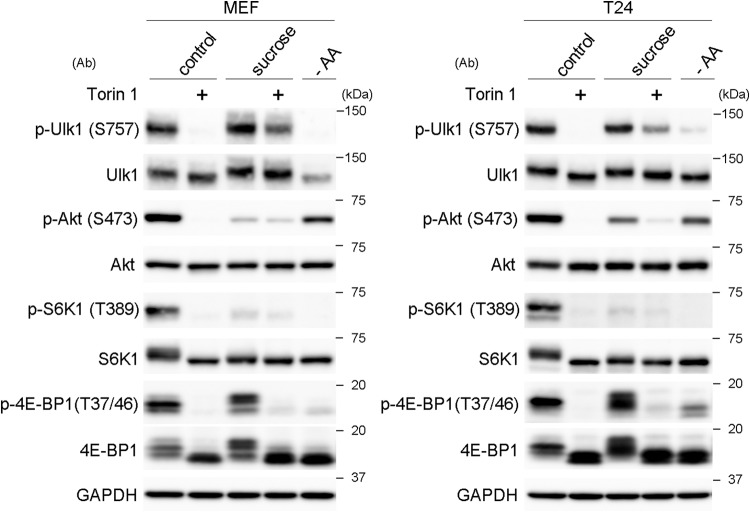
mTORC1 contributes to the phosphorylation of Ulk1 at S757 under hyperosmotic stress. Wild-type MEFs and T24 cells cultured for 1 h in DMEM with DMSO (control) or 1 μM torin 1 (+) were further cultured for 1 h in DMEM with 0.8 M sucrose containing the same reagents. The cells were also cultured in amino acid-free DMEM (−AA) for 1 h. The cell lysates were analyzed for Western blotting using antibodies against phosphorylated Ulk1 (S575), Ulk1, phosphorylated Akt (S473), Akt, phosphorylated S6K1 (T389), S6K1, phosphorylated 4E-BP1 (T37/46), 4E-BP1, and GAPDH as a loading control. Molecular weights are indicated on the right.

To better understand why p-Ulk1 remained unchanged by treatment with nonionic osmolytes, we used the mTOR inhibitor torin 1, which was expected to block the pathways of both mTORC1 and mTORC2. As expected, torin 1 apparently suppressed the phosphorylation of Ulk1, Akt, S6K1, and 4E-BP1 under normal conditions, and similar reductions were also observed under amino acid deprivation, except that the decrease of p-Akt was not so remarkable (Fig. 6). Under conditions of sucrose stress, torin 1 partially suppressed the phosphorylation of all the proteins, albeit to different extents (Fig. 6). Moreover, the phosphorylation of Akt, a substrate of mTORC2, was remarkably reduced under sucrose stress (Fig. 6), indicating that mTORC2 is suppressed under hyperosmotic stress. These results suggest that the high phosphorylation states of Ulk1, as observed under hyperosmotic stress, are mediated by multiple signaling mechanisms that involve mTORC1.

### The Ulk1 complex is not essential for hyperosmotic-stress-induced autophagy.

**FIG 7:**
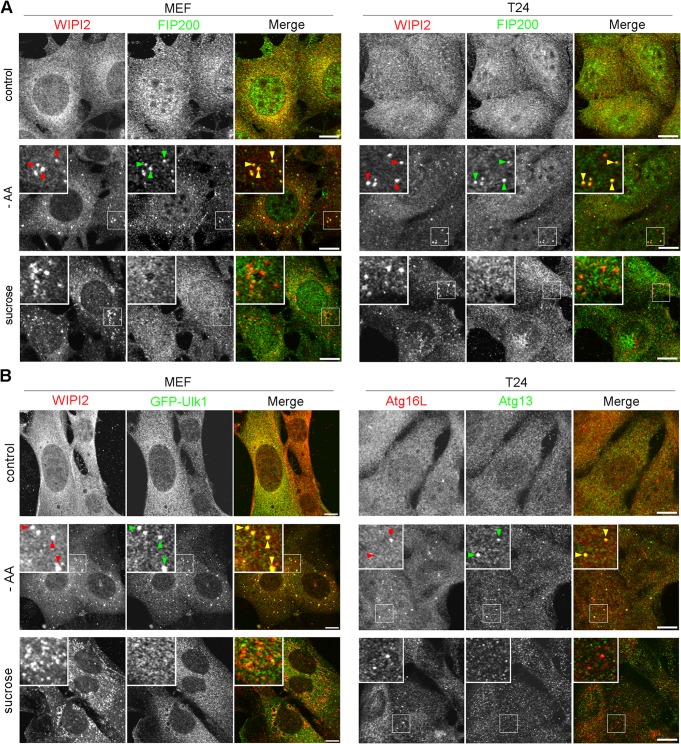
Ulk1 complex does not nucleate under hyperosmotic stress. (A) Wild-type MEFs and T24 cells were cultured in DMEM (control) or amino acid-free DMEM (−AA) for 120 min or with 0.8 M sucrose for 30 min. They were fixed and double immune labeled for WIPI2 (red) and FIP200 (green). The boxed regions are magnified in the insets. The yellow arrowheads indicate puncta double positive for WIPI2/FIP200. Bars, 10 μm. (B) Wild-type MEFs expressing GFP-Ulk1 (green) and T24 cells were cultured as for panel A. They were fixed and immunolabeled for WIPI2 (red) in MEFs and double immunolabeled for Atg16L (red) and Atg13 (green) in T24 cells. The boxed regions are magnified in the insets. The yellow arrowheads indicate puncta double positive for WIPI2/GFP-Ulk1 in MEFs and Atg16L/Atg13 in T24 cells. Bars, 10 μm.

**FIG 8:**
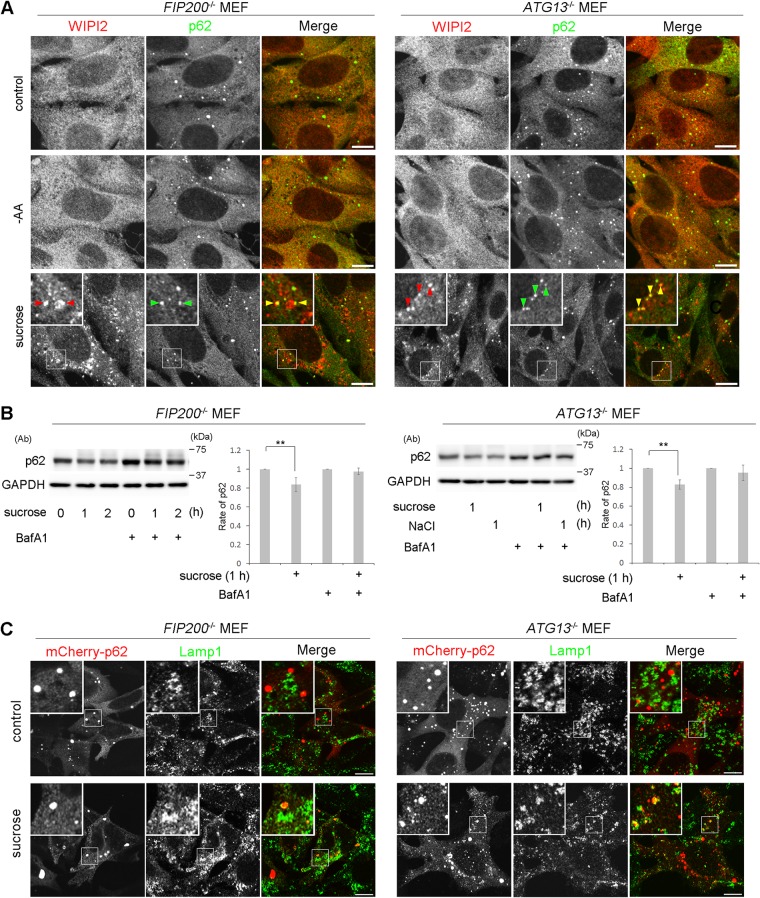
The Ulk1 complex is not essential for hyperosmotic-stress-induced autophagy. (A) *FIP200*^−/−^ MEFs and *Atg13*^−/−^ MEFs were cultured in DMEM (control) or amino acid-free DMEM (−AA) for 120 min or with 0.8 M sucrose for 30 min. They were fixed and double immune labeled for WIPI2 (red) and p62 (green). The boxed regions are magnified in the insets. The yellow arrowheads indicate puncta double positive for WIPI2/p62. Bars, 10 μm. (B) *FIP200*^−/−^ MEFs and *Atg13*^−/−^ MEFs were cultured with or without 100 nM bafilomycin A1 for 16 h and then with 0.8 M sucrose or 0.4 M NaCl for 1 h or 2 h, as indicated. The lysates were analyzed for Western blotting using antibodies against p62 or GAPDH as a loading control. Molecular weights are indicated on the right. The experiments were repeated 5 times. Band intensities were quantified, and ratios to the control value are plotted on the right. Statistical significance was determined by Student's *t* test. **, *P < *0.01. (C) *FIP200*^−/−^ MEFs and *Atg13*^−/−^ MEFs were transfected with mCherry-p62 and then cultured in DMEM (control) or DMEM with 0.8 M sucrose for 1 h. The cells were fixed and immunolabeled for Lamp1. The boxed regions are magnified in the insets. Bars, 10 μm.

**FIG 9:**
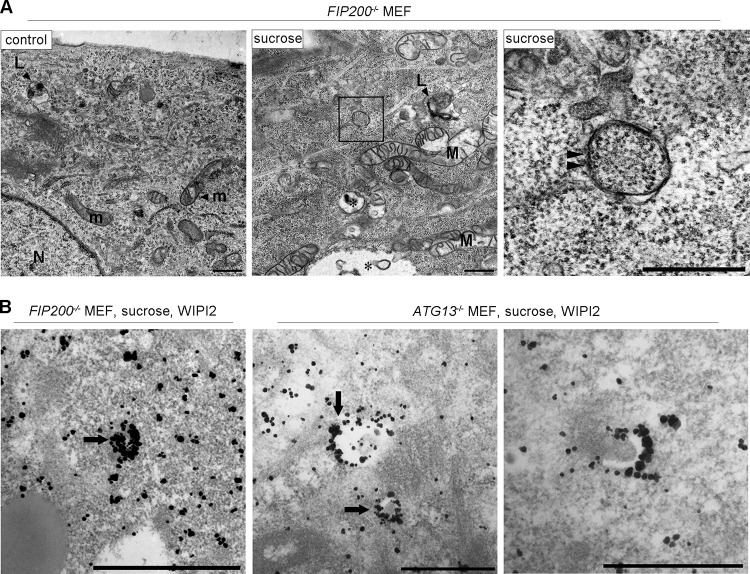
Electron microscopic analyses for autophagosomal structures and WIPI2 localization in *FIP200*^−/−^ or *Atg13*^−/−^ MEFs under hyperosmotic stress. (A) *FIP200*^−/−^ MEFs were cultured in DMEM with or without (control) 0.8 M sucrose for 1 h and then fixed for EM. The boxed region in the middle is shown enlarged on the right. (B) *FIP200*^−/−^ MEFs and *Atg13*^−/−^ MEFs were cultured with 0.8 M sucrose for 30 min and then fixed for immuno-EM using anti-WIPI2 antibody. Note that small autophagosome/IM-like profiles with double membranes (panel A, right, arrowheads), and those labeled with silver-enhanced particles indicating WIPI2 were detected. Asterisk, large endosome-like structures; L, lysosome-like profiles; m, mitochondria; M, degenerated mitochondria; N, nucleus. Bars, 0.5 μm.

The above-mentioned biochemical results prompted us to examine if the Ulk1 complex nucleates upon hyperosmotic stress in MEFs and T24 cells. By immunofluorescence microscopy, although endogenous FIP200, a component of the Ulk1 complex, appeared as cytoplasmic puncta with WIPI2 under amino acid deprivation, such nucleation was not observed with 0.8 M sucrose (Fig. 7A). Two other components, Atg13 and Ulk1, also did not form cytoplasmic puncta with 0.8 M sucrose (Fig. 7B). Because these results suggested that the Ulk1 complex is not essential for hyperosmotic-stress-induced autophagy, we next examined *FIP200*- or *Atg13*-deficient MEFs, in which conventional macroautophagy has been shown to cease (26, 27). Intriguingly, apparent punctate structures for WIPI2 were induced in these cells after hyperosmotic stress, while the proteins showed a diffuse pattern under amino acid deprivation (Fig. 8A). Moreover, a fraction of the puncta contained p62 (Fig. 8A), and flux assays with p62 revealed that hyperosmotic-stress-induced autophagy occurred in the mutant cells (Fig. 8B). In addition, both knockout MEFs showed the translocation of mCherry-p62 into lysosomes under hyperosmotic conditions (Fig. 8C). By EM, small autophagosome/IM structures were observed in *FIP200*-deficient MEFs (Fig. 9A), and these structures were decorated by WIPI2 in *FIP200*- or *Atg13*-deficient MEFs, as observed by immuno-EM (Fig. 9B).

However, in flux assays, the relative level of p62 that remained after 1 h of hyperosmotic stress was slightly higher in *FIP200*^−/−^ MEFs (0.84 ± 0.07) and Atg13^−/−^ MEFs (0.83 ± 0.05) (Fig. 8B) than in wild-type MEFs (0.73 ± 0.08) (Fig. 4A), and incorporation of mCherry-p62 into Lamp1-positive lysosomes appeared incomplete (Fig. 8C) compared with wild-type MEFs (Fig. 4E). These observations suggest that hyperosmotic-stress-induced autophagy was slowed in the mutant cells. Taking the data together, we conclude that the Ulk1 complex is not essential for hyperosmotic-stress-induced autophagy.

## DISCUSSION

### Hyperosmotic stress induces Ulk1-independent autophagy.

Previous studies have demonstrated that hyperosmotic stress induces acute autophagy in mammalian cells, primarily based on the induction of puncta for autophagosome markers and increased autophagy flux after the stress (10, 20, 28, 29). This conclusion has also been supported by another line of studies regarding the mTOR pathway, where hyperosmotic stress with nonionic osmolytes has been shown to suppress mTOR while activating AMPK (20, 22–24). In the present study, we reevaluated this hyperosmotic-stress-induced autophagy using MEFs and T24 cells and unexpectedly revealed that this unique type of autophagy proceeds independently of the Ulk1 complex, which is considered essential for the initiation of starvation-induced macroautophagy (30, 31). However, it should be noted that autophagy flux and p62 incorporation into lysosomes appeared to be delayed or incomplete in *FIP200*^−/−^ MEFs and *Atg13*^−/−^ MEFs compared with wild-type MEFs. Therefore, we cannot exclude the possibility that the Ulk1 complex contributes to the efficiency of hyperosmotic-stress-induced autophagy.

Interestingly, there have been other studies of Ulk1-independent autophagy in mammalian cells, including LC3-associated phagocytosis (LAP), ammonia-induced autophagy, and endosomal microautophagy. LAP is a phenomenon in which LC3 is transiently recruited on the phagosome membranes of macrophages. While this process is not dependent on Ulk1, it is dependent on the PI3K complex and ATG5/ATG7. Although this mechanism should be considered a nonautophagic function of the autophagy machineries, the results of these studies indicate that LC3 can be recruited to membranes independently of Ulk1 (32, 33). As for ammonia-induced autophagy, Cheong et al. suggested that autophagy responds not only to amino acid deprivation but also to nitrogen excess, which is caused by the accumulation of ammonia from prolonged glucose deprivation (∼24 h). The authors demonstrated that Ulk1/Ulk2 are not required for this type of autophagy (34). Very recently, endosomal microautophagy has been proposed; Mejlvang et al. have suggested that selective autophagy receptors, including p62 and NcoA4, can be rapidly (within 4 h) degraded via endosomes after amino acid deprivation, which is ESCRT dependent but independent of the conventional macroautophagy pathway involving mTOR (35). Although the presence of WIPI2 on large endosomal structures in immuno-EM seems to support the occurrence of acute degradation of p62/NcoA4 by this microautophagy mechanism under hyperosmotic conditions, we consider this unlikely because no profile for membrane invagination has been detected and WIPI2 was also localized to the small, typical autophagosome/IM profiles. Thus far, we do not know the function of WIPI2 in this endosomal fraction. Together, the previous studies support the presence of Ulk1-independent autophagy, and we propose that hyperosmotic-stress-induced autophagy is one example.

Recent studies have proposed a model of p62-mediated phase separation in which p62 forms large condensations harboring ubiquitinated proteins, thus potentially providing a scaffold for selective autophagy (36–38). We consider that this mechanism may be integrated into hyperosmotic-stress-induced autophagy for the elimination of ubiquitinated proteins. Indeed, polyubiquitinated proteins accumulate under hyperosmotic stress and form p62/ubiquitin-positive aggregates (10, 19). A key observation in our results would be the formation of cytoplasmic WIPI2/Atg16L puncta during hyperosmotic stress that was dependent on PI3K activity. Therefore, we speculate that unknown mechanisms may bypass Ulk1 signaling for PI3K-dependent IM formation near p62 condensation. Additional studies addressing this issue are warranted.

### mTOR kinase activities are modified under hyperosmotic stress.

Several studies have reported that mTORC1 activity is inhibited by hyperosmotic stress (20, 23, 24, 39, 40). This suggestion is also supported by reductions of p-S6K1 observed in our study. However, it remains controversial (24, 41, 42). Intriguingly, Kwak et al. have shown, using *in vitro* assays, that hyperosmotic stress increases mTOR activity. They also observed reduced phosphorylation of S6K1 and 4E-BP1, which was attributed to calyculin A-sensitive phosphatases (24). In this study, we showed that Ulk1 and 4E-BP1 apparently remained phosphorylated and that mTOR itself was also phosphorylated at S2448 under hyperosmotic stress with nonionic osmolytes. Therefore, it may be better to conclude that the kinase properties of mTOR are altered under hyperosmotic stress, thereby reflecting the phosphorylation states of its substrates. However, other mechanisms should be considered, including those related to other kinases and/or phosphatases and to the water outflux and influx that occur during hyperosmotic stress. As for Ulk1 phosphorylation, we demonstrated, by using n mTOR inhibitor, torin 1, that mTOR activity partially contributes. Furthermore, given that mTORC2 activity was shown to be remarkably reduced under hyperosmotic stress, mTORC1 most likely phosphorylates Ulk1 under hyperosmotic conditions. However, because the extent of Ulk1 dephosphorylation by torin 1 was partial, it may also be possible that other kinases or mechanisms are involved. More investigations are required to better understand the precise upstream mechanisms of both mTOR and Ulk1 under hyperosmotic-stress conditions.

## MATERIALS AND METHODS

### Antibodies and reagents.

Anti-WIPI2 was purchased from Abcam (catalog no. ab105459; IF, 1:400; immuno-EM, 1:400). Anti-Atg16L (catalog no. PM040; IF, 1:400) was purchased from MBL. Anti-Atg13 (catalog no. MABC46; IF, 1:200) was purchased from Merck Millipore. Anti-p62 was purchased from Progen (catalog no. GP62-C; IF, 1:800; WB, 1:1,000). Anti-GAPDH (anti-glyceraldehyde-3-phosphate dehydrogenase) (catalog no. sc-32233; WB, 1:2,000) and anti-Lamp1 (catalog no. sc-19992; IF, 1:400) were purchased from Santa Cruz. Anti-NcoA4 (ARA70) was purchased from Bethyl Laboratories (catalog no. A302-272A; WB, 1:500). Anti-FIP200 (RB1CC1) was purchased from Proteintech (catalog no. 17250-1-AP; IF, 1:400). Anti-mTOR (catalog no. 2983; WB, 1:1,000), anti-phospho-mTOR Ser2448 (catalog no. 5536; WB, 1:1,000), anti-Ulk1 (catalog no. 8054; WB, 1:1,000), anti-phospho-Ulk1 Ser757 (catalog no. 6888; WB, 1:1,000), anti-S6K1 (catalog no. 9202; WB, 1:1,000), anti-phospho-S6K1 Thr389 (catalog no. 9206; WB, 1:1,000), anti-4E-BP1 (catalog no. 9644; WB, 1:1,000), anti-phospho-4E-BP1 T37/46 (catalog no. 2855; WB, 1:1,000), anti-Akt (catalog no. 9272; WB, 1:1,000), anti-phospho-Akt S473 (catalog no. 4060; WB, 1:1,000), and anti-hVps34 (catalog no. 3811; WB, 1:1,000) were purchased from Cell Signaling Technology. Mouse secondary antibodies conjugated with Alexa Fluor 488 (IF, 1:800) and 594 (IF, 1:800), rabbit secondary antibodies conjugated with Alexa Fluor 488 (IF, 1:800) and 594 (IF, 1:800), and guinea pig secondary antibodies conjugated with Alexa Fluor 647 (IF, 1:800) were purchased from Jackson ImmunoResearch. Mouse secondary antibody conjugated with horseradish peroxidase (HRP) (catalog no. NA931; WB, 1:2,000) and rabbit secondary antibody conjugated with HRP (catalog no. NA934; WB, 1:2,000) were purchased from GE Healthcare. Protein A conjugated with HRP (catalog no. 101023; WB, 1:2,000) was purchased from Thermo Fisher Scientific. Nanogold-labeled anti-mouse Fab′ was purchased from Nanoprobes (catalog no. 2002; used at 1:100 for immuno-EM). The chemicals used in this study were bafilomycin A1 (final concentration, 100 nM; Merck Millipore), E-64-d (final concentration, 10 μg/ml; Peptide Institute, Inc.), pepstatin A (final concentration, 10 μg/ml; Peptide Institute, Inc.), epoxomicin (final concentration, 5 μM; Wako), lactacystin (final concentration, 10 μM; Peptide Institute, Inc.), torin 1 (final concentration, 1 μM; ChemScene), Vps34-IN1 (final concentration, 200 nM; Cayman Chemical), and wortmannin (final concentration, 1 μM; Sigma-Aldrich). siRNA oligonucleotides were purchased from Ambion (Thermo Fisher Scientific; control siRNA, catalog no. AM4611; hVPS34 siRNA, catalog no. s10517).

### Plasmids.

A cDNA fragment encoding mouse Ulk1 was amplified from the genomes of wild-type MEFs with forward (5′-AAGGGATCCGAATTCATGGAGCCGGGCCGCGGCGGCG-3′) and reverse (5′-AGATGCATGCTCGAGTCAGGCATAGACACCACTCAGCAG-3′) primers. It was cloned into EGFP-pcDNA at EcoRI-XhoI sites, resulting in GFP-Ulk1-pcDNA. A cDNA for GFP-Ulk1 was amplified from GFP-Ulk1-pcDNA with forward (5′-ATTTCCGGTGAATTCATGGTGAGCAAGGGCGAG-3′) and reverse (5′-GGTAGAATTGGATCCTCAGGCATAGACACCACTCAGCAG-3′) primers and was cloned into pLVSIN-CMV-puro (TaKaRa), resulting in GFP-Ulk1-pLVSIN-CMV-puro. To generate pmCherry-p62, p62 was amplified by PCR and digested with EcoRI-XhoI. The PCR product was subcloned into the EcoRI-SalI site of the pmCherry-C2 vector (Clontech, Mountain View, CA).

### Cell culture, transfections, and generation of a stable cell line.

T24 cells (catalog no. JCRB0711) were purchased from the JCRB cell bank (National Institutes of Biomedical Innovation, Japan). *Atg13*^−/−^ MEFs were a gift from N. Mizushima (26), and *FIP200*^−/−^ MEFs were a gift from J. L. Guan (43). MEFs and T24 cells were cultured in high-glucose Dulbecco’s modified Eagle’s medium (DMEM) (Nacalai; catalog no. 08458-16) supplemented with 9.09% fetal bovine serum (FBS) (Sigma-Aldrich; catalog no. 171021; lot no. 14K525). To induce starvation, the cells were incubated in amino acid-free medium (Wako; catalog no. 048-33575) for 30 min, 1 h, or 2 h. To induce hyperosmotic stress, the cells were shifted to DMEM supplied with the indicated concentration of sugars or salts. The mCherry-p62 plasmid was transfected into cells with the FuGene HD transfection reagent (Promega) for 48 h. The cells were transfected with siRNA oligonucleotides (final concentration, 33.3 nM) using Lipofectamine RNAi Max transfection reagent (Thermo Fisher Scientific) for 72 h. Stable GFP-Ulk1-expressing MEFs was selected in 10 μg/ml puromycin (InvivoGen).

### Cell viability.

Cells grown in DMEM were cultured in DMEM with or without 0.8 M sucrose for 1 h. After changing the medium to DMEM, they were further cultured for 0, 24, or 48 h, followed by procedures of fixation and staining with Hoechst 33342 (Thermo Fisher Scientific). Images were acquired with a fluorescence microscope (Olympus; BX51).

### Immunoblotting.

Cells were washed in cold phosphate-buffered saline (PBS) once and lysed in PBS with 1% SDS. The lysates were immediately boiled for 7 min. After sonication (Tomy; catalog no. UR-20P), 5 to 10 μg of each total lysate was loaded into 5% to 20% gradient gels (Wako; catalog no. 194-15021) and transferred to polyvinylidene difluoride (PVDF) membranes (Merck Millipore; catalog no. IPVH00010). The blots were blocked with 5% skim milk in phosphate-buffered saline containing 0.1% Tween 20 (PBST) and probed with the indicated antibodies.

### Quantification of immunoblotting.

The band intensity was analyzed by ImageQuant TL (GE Healthcare). Each value was normalized by the expression level of GAPDH and then compared with control samples. At least three independent experiments were performed. Statistical significance was determined by Student´s *t* test.

### Immunofluorescence microscopy.

MEFs and T24 cells grown on coverslips were fixed with 4% paraformaldehyde in PBS for 15 min and permeabilized with 0.1% Triton X-100 in PBS for 20 min. The cells were blocked in PBST containing 0.4% bovine serum albumin (BSA) for 30 min and then incubated with primary antibodies in PBST. The coverslips were incubated with secondary antibodies in PBST for 1 h. Images were acquired with an FV1000 (Olympus) confocal microscope equipped with a PlanApo N 60× (numerical aperture [NA], 1.42; oil) or a UPlanApo 100× (NA, 1.40; oil) lens.

### Electron microscopy.

MEFs and T24 cells grown on coverslips were fixed in 0.1 M phosphate buffer (pH 7.4) with 2% paraformaldehyde and 2% glutaraldehyde, followed by treatment with 1% osmium tetroxide and 1.5% potassium ferrocyanide in 0.1 M phosphate buffer (pH 7.4) for 1 h. The cells were dehydrated in ethanol and incubated in propylene oxide. They were then embedded in epoxy resin and sectioned. For immuno-EM, cells were fixed in 0.1 M phosphate buffer (pH 7.4) containing 4% paraformaldehyde with or without 0.01% glutaraldehyde for 20 min. The cells were permeabilized with 0.02% saponin for 20 min, blocked in PBS buffer with 0.1% BSA and 0.005% saponin, and incubated with WIPI2 antibody overnight. Subsequently, the coverslips were incubated with secondary antibodies overnight. The signals were enhanced with an HQ Silver enhancement kit (Nanoprobes, Inc.), followed by treatment with 0.5% osmium tetroxide in 0.1 M phosphate buffer (pH 7.4) for 90 min. They were then embedded in resin as described above. Images were obtained with an electron microscope (JEM1200EX; JEOL).
